# Public Pharmacists' Association

**Published:** 1920-11-06

**Authors:** 


### Public Pharmacists' Association.

The opening meeting of the session was recently held at St. Bride Institute, London. Mr. J. L. P. Hollingworth, F.L.S., M.P.S. (Chief Pharmacist, Victoria Hospital, Chelsea), in the chair, presided over a good gathering of pharmacists engaged at hospitals and institutions. Various business matters were dealt with, after which the evening was devoted to hearing three "short papers," followed by discussion.

The Council decided to delete the usual formal address for the opening meeting, and the innovation was a decided success.

Mr. R. Welford spoke on the "Future of Pharmacy," and prophesied a bright future for pharmacists, provided that, after qualifying, they kept themselves informed of the newer remedies, etc., so that they would be ready to meet the requirements of the medical officers attached to their institution.

Mr. Henry Rodwell (St. Thomas's Hospital) gave a paper on "Should Public Pharmacists Manufacture?" As the head of the manufacturing laboratory at a large hospital, Mr. Rodwell's experience entitles him a claim for knowledge on the subject; he advocated, wherever possible, manufacturing all preparations it was possible to do, both from the standpoint of economy and purity.

The author pointed out the difficulty of proving by analysis the purity of certain galenical preparations unless one had examined the drugs, etc., used when in the crude state. This paper gave rise to a good discussion. Mr. Jenkin expressed the view that manufacturing only paid if machinery was in use fairly regularly and large quantities made.

Mr. J. Heworth gave his experiences as "Seven Years as a Locum," and detailed the various methods of carrying on hospital dispensaries where he had acted. Altogether the evening proved a very profitable one, and members expressed their thanks for the arrangements made enabling them to gain valuable information.

